# Eye-Tracking Reveals a Role of Oxytocin in Attention Allocation Towards Familiar Faces

**DOI:** 10.3389/fendo.2021.629760

**Published:** 2021-05-17

**Authors:** Nina Marsh, Dirk Scheele, Danilo Postin, Marc Onken, Rene Hurlemann

**Affiliations:** ^1^ Department of Psychiatry, School of Medicine and Health Sciences, Carl von Ossietzky University Oldenburg, Oldenburg, Germany; ^2^ Department of Psychiatry, University Hospital Bonn, Bonn, Germany; ^3^ Research Center Neurosensory Science, Carl von Ossietzky University Oldenburg, Oldenburg, Germany

**Keywords:** oxytocin, eye-tracking, familiar faces, visual attention, eye region, autistic-like traits

## Abstract

Visual attention directed towards the eye-region of a face emerges rapidly, even before conscious awareness, and regulates social interactions in terms of approach versus avoidance. Current perspectives on the neuroendocrine substrates of this behavioral regulation highlight a role of the peptide hormone oxytocin (OXT), but it remains unclear whether the facilitating effects of OXT vary as a function of facial familiarity. Here, a total of 73 healthy participants was enrolled in an eye-tracking experiment specifically designed to test whether intranasal OXT (24 IU) augments gaze duration toward the eye-region across four different face categories: the participants’ own face, the face of their romantic partner, the face of a familiar person (close friend) or an unfamiliar person (a stranger). We found that OXT treatment induced a tendency to spend more time looking into the eyes of familiar persons (partner and close friend) as compared to placebo. This effect was not evident in the self and unfamiliar conditions. Independent of treatment, volunteers scoring high on autistic-like traits (AQ-high) spent less time looking at the eyes of all faces except their partner. Collectively, our results show that the OXT system is involved in facilitating an attentional bias towards the eye region of familiar faces, which convey safety and support, especially in anxious contexts. In contrast, autistic-like traits were associated with reduced attention to the eye region of a face regardless of familiarity and OXT-treatment.

## Introduction

Visual attention towards a face at the initial stages of interpersonal communication emerges outside conscious awareness and deliberate control and regulates social interactions in terms of approach and avoidance. Individuals rely on faces to identify individuals and extract socially relevant information such as gender, age and emotional state. Specifically, attention to the eye region seems to be highly relevant due to its dual function in human social interaction: individuals can both perceive information from the eye region of others and use their eye gaze to signal information to others (1). Many neuropsychiatric disorders, including autism spectrum disorder (ASD), are characterized by impairments in visual attention to and facial assessment of a social counterpart, which may lead to difficulties in establishing social relationships (2, 3). Most studies examining the patterns of gaze to faces in individuals diagnosed with ASD reveal reduced attention to the central features of the face, and especially the eye region of a face (4), which may reflect diminished social motivation (5).

Current perspectives on the neurobiology of visual face processing suggest that it is deeply rooted in the neuroendocrine architecture of the social brain. The hypothalamic neuropeptide hormone oxytocin (OXT) modulates a broad repertoire of social behaviors, including social communication, interpersonal trust, and prosocial decision-making (6–8) in a person-specific and context-dependent manner (9). An extensive body of research supports the idea that OXT influences early stages of social information processing (10–12). For example, in non-human primates, OXT increases the time that rhesus monkeys spend viewing the eyes of conspecifics in static images (13, 14). Furthermore, the peptide enhances socially reinforced learning (15), and increases reward allocation to a partner monkey (16). In humans, the peptide facilitates the memory of facial identity (17), biases the processing of facial valence by enhancing the encoding of happy faces (18), and decreases the aversion towards angry faces (19). Mechanistically, these effects may result from OXT acting upon visuo-cognitive mechanisms within the core face-processing system (20) – an assumption that is further substantiated by findings from neuroimaging studies (11, 21). Alternatively, OXT may promote face processing by increasing the sensitivity to socially salient cues (22, 23). Evidence for this account was provided by findings showing that OXT increases attentional shifts toward emotional cues (24, 25) as well as the gaze duration towards the eye region (26, 27). This is further supported by studies showing that OXT shifts altruistic priorities towards a social charity project at the cost of an environmental charity project (28), and that OXT’s role in promoting cooperation critically depends on the presence of social information (29). With previous neuroimaging studies showing that OXT consistently targets reward- [(30–32), but see (33)] and fear-related neurocircuits (34–37), the peptide possibly facilitates attention to social stimuli by modulating the rewarding experience from interpersonal interactions (18, 38), and this experience may be particularly pronounced during encounters with familiar others. In this context, however, it is important to note, that the effects of OXT are highly susceptible to individual personality traits and situational variables (9, 39). For example, it has been found that OXT increases envy and gloating (40), decreases the tendency to cooperate in individuals with borderline personality disorder (41), and selectively increases the pleasantness of interpersonal touch in individuals scoring low in autistic-like traits (42). Given that OXT does not exclusively promote positive social behaviors across all individuals and across all situations, the peptide may increase visual attention to the eye region of faces only under certain conditions. While prior studies investigated the effects of OXT on the ability to recognize differences between self and others (43), emotion expression (44), and gaze toward the eye-region of neutral faces (26), it remains unclear whether the facilitating effects of OXT vary as a function of facial familiarity. New insights on the modulatory role of OXT in the context of facial familiarity would be informative given that familiar faces convey safety and support, especially in anxious contexts. In the present study, we used eye-tracking to assess visual attention to the eye-region of faces involving a total of 73 pair-bonded participants. After treatment with 24-IU OXT or placebo (PLC), the participants viewed personalized, dynamic video clips, which involved four different categories of faces: the participants own face, the face of their romantic partner, the face of a familiar person (close friend) or an unfamiliar person (a stranger).

## Materials and Methods

### Participants

A total of 73 healthy, pair-bonded female (n = 45) and male (n = 28) volunteers (mean age ± SD: 24.53 ± 5.20 years) were enrolled in the study after giving written, informed consent. Female participants completed a pregnancy test to confirm that they were not pregnant. Subjects were free of current and past physical or psychiatric illness. All subjects were in a heterosexual relationship for more than 6 months, had no children and had normal or corrected-to-normal vision. Moreover, subjects were naive to prescription-strength psychoactive medication and had not taken any over-the-counter psychoactive medication in the preceding four weeks. Subjects were asked to maintain their regular sleep and waking times and to abstain from caffeine and alcohol intake on the day of the test session.

### Procedure

We used a double-blind, randomized parallel-group trial design and administered a 24-IU nasal dose of either synthetic OXT^IN^ or placebo (PLC^IN^), both provided by Sigma-Tau Pharmaceuticals, Inc. (Pomezia, Italy). The placebo solution contained the identical ingredients except the peptide itself. The experiment comprised an initial screening session followed by the test session one week apart. Screening entailed the exclusion of current or past physical or psychiatric illness (including drug and alcohol abuse) as assessed by medical history and the Mini-International Neuropsychiatric Interview (MINI) (45). To control for possible pre-treatment differences, we assessed current state and trait anxiety with the State Anxiety Inventory (46), depressive symptoms with the Beck Depression Inventory (BDI) (47), alexithymia with the Toronto Alexithymia Scale (TAS) (48), autistic-like traits with the Autism-Spectrum-Quotient (AQ) (49). Furthermore, possible mood changes were assessed using the Positive and Negative Affect Scale [PANAS (50)] before and after the experimental task. There were no a-priori differences between the OXT^IN^ and PLC^IN^ groups on these factors (**Table 1**).

**Table 1:** Demographics, personality traits, and attitudes.

	OXT group	PLC group	*t*	*P*
	Mean (SD)	Mean (SD)		
Age (years)	24.80 (5.43)	24.29 (5.04)	-0.41	.67
Education (years)	16.67 (2.98)	16.08 (2.57)	-0.90	.37
Autism-spectrum Quotient (AQ)	16.11 (6.92)	14.15 (6.58)	-1.26	.21
Beck-Depression Inventory (BDI)	4.14 (3.91)	2.61 (2.90)	-1.91	.06
State-Trait Anxiety Inventory (STAI-Trait)	36.11 (8.66)	32.71 (7.89)	-1.75	.08
State-Trait Anxiety Inventory (STAI-State)*	33.77 (5.19)	34.10 (5.82)	0.25	.79
State-Trait Anxiety Inventory (STAI-State)**	32.37 (5.69)	30.78 (5.98)	-1.15	.25
Positive-Negative Affect Schedule (PANAS, positive)*	32.42 (5.93)	30.76 (5.32)	-1.26	.21
Positive-Negative Affect Schedule (PANAS, negative)*	12.14 (2.62)	11.34 (1.64)	-1.57	.12
Positive-Negative Affect Schedule (PANAS, positive)**	32.00 (7.66)	30.71 (7.55)	-0.72	.47
Positive-Negative Affect Schedule (PANAS, negative)**	10.88 (1.85)	10.50 (1.22)	-1.03	.30
Marburger Einstellungs-Inventar f. Liebesstile (MEIL)	5.28 (0.48)	5.24 (0.58)	-0.27	.78
	Absolute	Absolute	***χ²***	*P*
Gender (female/male)	23/12	22/16	1.00	.75

OXT, oxytocin; PLC, placebo; *pre-task measurement; **post-task measurement; SD, standard derivation.

**Figure 1:**
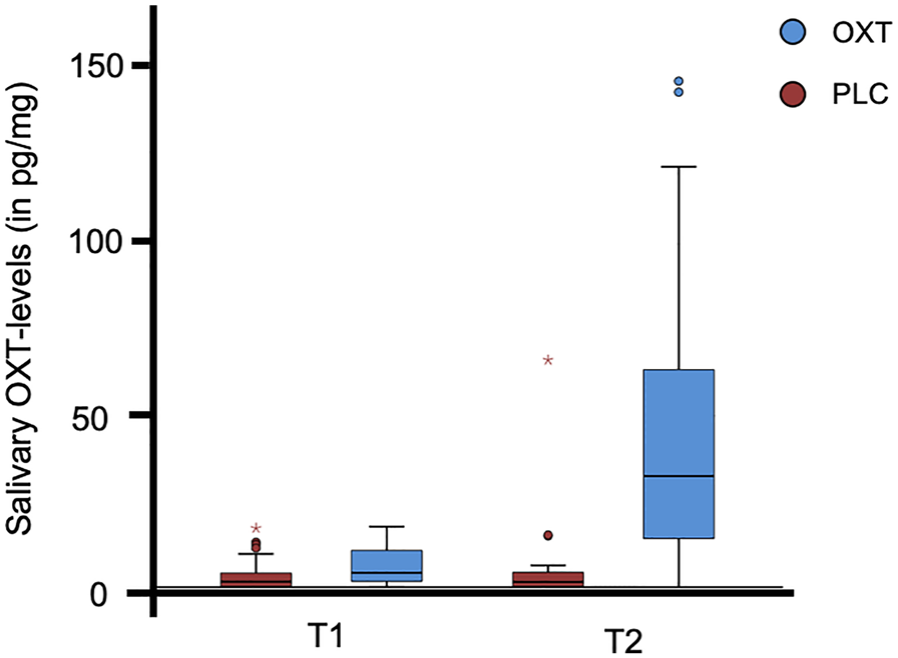
The distribution of salivary oxytocin (OXT) levels for the participants (N = 70). The box plot illustrates the data for both the OXT and the placebo (PLC) group prior to the self-administration of the nasal spray (T1) and after completion of the experimental trial (T2). Three subjects were excluded from the analyses due to incomplete saliva samples. °, values >1.5 times of the interquartile range; * values >3.0 times of the interquartile range.

Before the self-administration of the nasal spray as well as after completion of the experimental task, participants were asked to submit a saliva sample. The distribution of salivary oxytocin is illustrated in **Figure 1** and **Table 2**. The data from three subjects were excluded from the analyses due to incomplete saliva samples. Saliva samples were collected with commercial sampling devices (Salivettes, Sarstedt) and Salivettes were immediately centrifuged at 4,180g for 2 min and stored at -80°C until assayed. Saliva OXT was extracted and quantified by a highly sensitive and specific radioimmunoassay (51).

**Table 2:** Measures of salivary OXT-levels.

	OXT group (N = 34)	PLC group (N = 36)
	Mean (SD)	Mean (SD)
T1 (in pg/mg)	6.02 (5.24)	3.49 (4.47)
T2 (in pg/mg)	45.12 (40.08)	4.65 (11.15)

OXT, oxytocin; PLC, placebo; SD, standard derivation; T1, time of measure 1: prior to self-administration of the nasal spray; T2, time of measure 2: after completion of the experimental trial.

### Eye-Tracking Task

**Figure 2:**
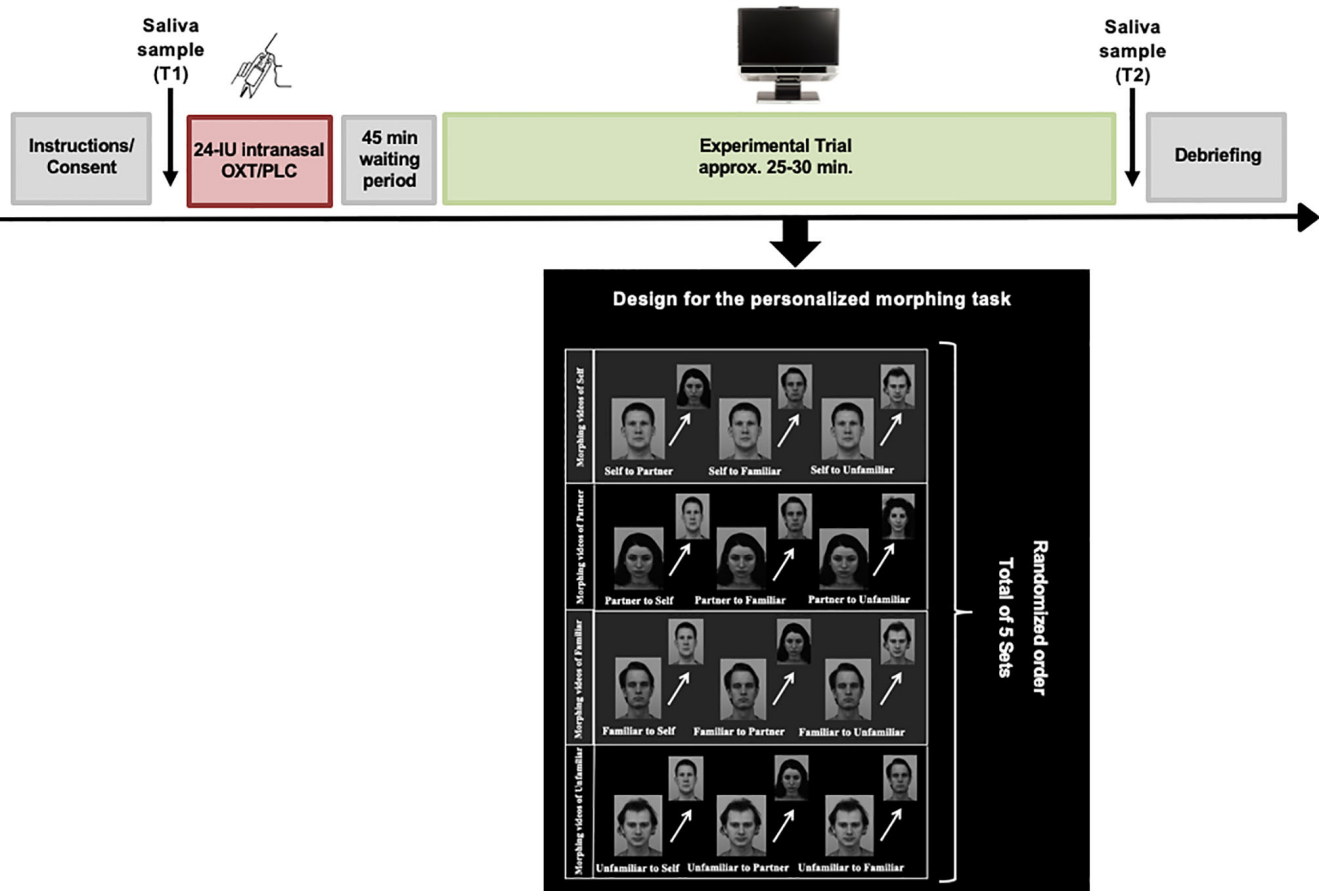
Overview of the experimental design. After intranasal administration of oxytocin or placebo spray and a waiting period of 45 minutes, the participants were seated in front of the eye-tracker. Following calibration, they were exposed to the experimental task consisting of twelve personalized morphing videos involving four categories of faces (“self”, “partner”, “familiar”, “unfamiliar”). A total of five sets each consisting of twelve individual videos were shown to the participants in randomized order. After each set, a fixation cross was presented to ensure that the eyes of the participants were in a neutral position on the screen. Saliva samples were collected prior to the self-administration of the nasal spray (T1) and after completion of the experimental trial (T2). In this figure, facial images from the Karolinska Directed Emotional Faces (KDEF) database. The image ID's AF15NES, BM11NES, BM13NES, BM21NES are used for this illustration purpose (52).

In order to create the personalized experimental trials, all participants were asked to submit four photographs of the following categories to the study team: the participants’ own face, the face of their romantic partner, the face of a familiar person (same-sex close friend) or an unfamiliar person (a stranger) prior to the experimental session. Additionally, an unfamiliar person with comparable demographics (e.g. age, gender and ethnicity) was matched with each of the photos submitted by the participants. The unfamiliar faces were selected from the Karolinska Directed Emotional Faces (KDEF) (52). All pictures were edited to black and white, and a series of morphing videos were constructed by interpolating the photo of the participants own face into their partner’s face (self to partner), the familiar face (self to familiar) and the unfamiliar face (self to unfamiliar) in steps of 5% change using Abrosoft FantaMorph 5 (www.fantamorph.com). Approximately one hundred points were set on equivalent spots of the faces (e.g. eyes, mouth, nose) to create a fine-grained transition from one face to another. Each morphing video lasted 6.6 seconds. This procedure was repeated with the photo of the partner, as well as the familiar and unfamiliar person, resulting in twelve personalized morphing sequences for each participant. One additional video of two unfamiliar faces was created for training purposes prior to the experimental session. The experiment was conducted using Tobii Studio eye-tracking software version 3.2.3. During the experimental session, participants were seated in front of a Tobii TX300 binocular eye-tracker with a 23-inch display, a maximum resolution of 1920 x 1080 pixels, 0.01° precision, and a sampling rate of 300 Hz (Tobii Technology, 2012). After calibrating the eyes of the participants, they were presented with an introductory slide followed by a practice trial using faces that were not presented in the experimental trial. During the experimental session a total of five sets each consisting of twelve personalized videos with all of the four stimulus types morphing in both directions, for example from self to partner and vice versa. The videos were presented to the participant in randomized order (**Figure 2**). The participants were instructed to press the space button on the keyboard of the computer as soon as they identified the face into which the person in the video was being transformed. Their answers were recorded *via* a questionnaire, which appeared directly after the participants stopped the morphing sequence by pressing the space button. For the data analyses, the starting picture of each video was used in order to assess the gaze duration. The time until the participants identified the person into which the starting picture was being transformed was considered as reaction time in the later analyses. After each set, a fixation-cross appeared in the middle of the screen to ensure that the eyes of the participants were in a neutral position. Prior to data analyses, areas of interest (AOIs) were drawn around the eyes, mouth, or other non-eye-or-mouth face areas using freeform shapes in Tobii Studio.

### Statistical Analysis

Demographic, neuropsychological, and behavioral data were analyzed using IBM SPSS Statistics 22 (IBM, New York, NY, USA). Main effects and interactions were identified *via* repeated measures analysis of variance (ANOVA) and paired t-tests. All reported p-values are two-tailed. Significance was considered in case of P-values of *P* <.05. Effect sizes are given as measures of Eta-squared and Cohen’s *d*. Based on the subjects’ AQ scores, the sample was median-dichotomized, resulting in n = 39 AQ-low scorers (AQ-score ≤ 15), and n = 34 AQ-high scorers (AQ-score > 15).

## Results

**Figure 3:**
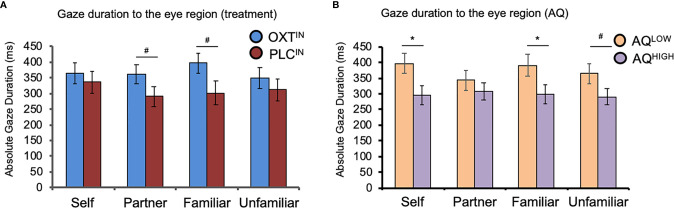
The effects of oxytocin (OXT) and autistic-like traits measured with the Autism-Spectrum-Quotient (AQ) on visual attention to the eye region of faces. **(A)** The effects of intranasal OXT on gaze duration toward the eye region across the four different categories of facial stimuli. Intranasal OXT increased the tendency to spend more time looking toward the eye region of the romantic partner (“partner”) and a close friend (“familiar”). **(B)** The effects of AQ on visual attention to the eye region across the four different categories of facial stimuli. Independent of treatment, individuals in the AQ-high group spent less time looking towards the eye region of their own face, as well as the familiar and unfamiliar person. OXT^IN^, oxytocin nasal spray; PLC^IN^, placebo nasal spray; AQ, Autism-Spectrum-Quotient; ms, milliseconds. Error bars indicate the standard error of the mean (SEM). ^#^ *P* < 0.10; *P < 0.05.

A mixed-design ANOVA with ‘familiarity’ (self, partner, familiar person, and unfamiliar person) as within-subject factor,’ treatment’ (OXT, PLC) and AQ (low autistic-like traits, high autistic-like traits) as between-subject variables, and the relative total gaze time toward the eye region as dependent variable showed a trend significant effect of treatment (*F*_(1, 63)_ = 3.58, *P* = 0.06,η^2^ = 0.05; **Figure 3A**) and a main effect of AQ (*F*_(1, 63)_ = 4.90, *P* = 0.03,η^2^ = 0.07; **Figure 3B**).

Participants in the OXT group spent more time looking in the eye region of faces. There was no significant main effect of familiarity or interaction between familiarity and treatment (all *P*’s > 0.05), but exploratory post-hoc t-tests revealed trend-significant effect such that OXT increased gaze to the eyes toward the face of the partner (*t*_(65)_ = 1.76, *P* = .08, *d* = 0.43) and close friends (*t*_(65)_ = 1.75, *P* = .09, *d* = 0.42). However, the OXT effect was not significant in the self (*P* = 0.65, *d* = 0.28) and unfamiliar conditions (*P* = 0.50, *d* = 0.16). Furthermore, an ANOVA with the additional factor gender did not reveal significant main or interaction effects of gender (*P* > 0.05). We found that, compared to AQ-low scorers, participants in the AQ-high group displayed a shorter gaze duration toward the eye region of their own photo (*t*_(66)_ = 2.38, *P* = .02, *d* = 0.59), the familiar person (*t*_(65.58)_ = 2.10, *P* = .04, *d* = 0.51), and a trend significant effect for the familiar person (*t*_(66)_ = 1.90, *P* = .06, *d* = 0.47), while there was no significant effect for the partner stimuli (*P* = 0.39). An additional repeated measures ANOVA with the within-subject factors ‘time’ (pre, post) and ‘treatment’ (OXT, PLC) and the salivary OXT concentration as dependent variable yielded main effects of time (*F*_(1, 71)_ = 37.15, *P* < 0.01,η^2^ = 0.34) and treatment (*F*_(1, 71)_ = 33.28, *P* < 0.01,η^2^ = 0.32) as well as an interaction of time and treatment (*F*_(1, 71)_ = 29.85, *P* < 0.01,η^2^ = 0.30). Post hoc t-tests revealed a significant increase in OXT concentration in participants treated with OXT (pre: 5.86 ± 5.26 pg/ml; post: 44.24 ± 40.01 pg/ml; *t_(34)_* = 5.83, *P* < 0.01, *d* = 1.34) but not in those who received PLC (pre: 3.37 ± 4.38 pg/ml; post: 5.46 ± 12.24 pg/ml; t_(37)_ =1.07, *P* = 0.29, *d* = 0.23). Neither the OXT baseline concentrations nor the difference before and after the task in the PLC group correlated with the eye gaze duration (all *P*s > 0.05). Gaze to the mouth and other regions of the face was not affected by OXT. Independent of OXT-treatment and AQ, the participants responded faster if the morphing video started with a photograph of an unfamiliar person (mean ± SD: 3604 ± 756 ms) than with the photograph of the partner (3795 ± 711 ms; *t*_(72)_ = -2.76, *P* < 0.01, *d* = -0.26), self (3789 ± 682 ms; *t*_(72)_ = -2.71, *P* < 0.01, *d* = -0.26), or familiar person (3806 ± 690 ms; *t*_(72)_ = -2.60, *P* = 0.01, *d* = -0.28).

## Discussion

This study investigated the modulatory effect of OXT on visual attention to the eye-region across four categories of faces. The results indicate that OXT induced a tendency to spend more time looking into the eyes of familiar faces, suggesting that the peptide may promote visual attention towards personal familiar faces but not to one’s own and an unfamiliar face. This finding is consistent with evidence emphasizing OXT’s modulation of neural reward circuits during the presentation of socially relevant cues (32, 53). As such, the modulatory effect of OXT on eye gaze seems to be more pronounced for familiar faces. A subjective experience of reward when seeing a familiar face may result from increased trust, comfort and safety for the viewer, which may produce synergistic effects in combination with OXT’s modulatory role on trust and social support (54, 55). This finding also corroborates the notion of the peptides’ susceptibility to diverse interindividual and situational factors, which may produce heterogeneity in behavioral effects of OXT in humans (7, 28, 56–58). In fact, a previous study found that OXT increased the eye focus across picture categories (e.g. parent-child dyads and romantic couples), but the study tested participants with and without a romantic relationship and did not include bonding-specific stimuli (59). Our results also add to the literature describing social-stimulus specific effects of OXT on feedback-guided learning (60), thus, providing further support for the modulatory effects of the neuropeptide on processing social stimuli in humans. Furthermore, we did not observe a gender-specific effect of OXT in this study, which might be due to the small size of our sample and/or the imbalanced gender distribution within our sample (female: n = 45; male: n = 28).

Independent of treatment we also found that individuals with high-autistic-like traits exhibited a shorter gaze duration toward the eye region of a face. This is consistent with previous research emphasizing that ASD is characterized by reduced attention specific face regions (61), and replicates findings reported in clinical assessments, where eye contact is substantially reduced in individuals with ASD (62, 63). Furthermore, this observation is in line with evidence suggesting that AQ-scores in individuals represent a predictor for gaze perception, although this finding was only observed in males (64). According to Tanaka & Sung (65) this gaze avoidance tendency in individuals with ASD is an adaptive and compensatory strategy that protects them from social threat and discomfort caused by direct eye contact. It should be emphasized, though, that the measured AQ scores represent relatively typical levels of autistic-like traits within the general population (66). Future research with a focus on clinical ASD is needed to provide a more nuanced understanding of the effects of OXT on visual attention toward familiar faces.

Given that only pair-bonded individuals where included in this study and those were limited to undergraduate students (mean age ± SD: 24.53 ± 5.20 years) who tend to perform reasonably well in such experimental paradigms. Thus, our results may not be generalizable to individuals who experience these tasks as more challenging (67). Furthermore, it should be acknowledged that there was no assessment of the menstrual cycle phase or the use of hormonal contraceptives in the female participants. Thus, we cannot rule out potential interactions of exogenous OXT with fluctuations of steroid hormones over the menstrual cycle (68–70). Future studies should therefore consider assessing the influence of OXT on the attention towards familiar faces by including participants of different age groups, with a different relationship status, and a more nuanced assessment of the female cycle stages. Although these factors may limit the generalizability of our findings, the present study contributes to a better understanding on how the oxytocinergic system is involved in visual attention towards the eye-region of familiar faces. More generally, our results corroborate the notion that the peptide’s effects on gaze behavior to human faces are context-dependent and that OXT is a complex neuromodulator, whose behavioral effects are highly susceptible to variation due to individual and contextual variables.

## Data Availability Statement

The raw data supporting the conclusions of this article will be made available by the authors upon request, without undue reservation.

## Ethics Statement

The study was approved by the Institutional Review Board of the Medical Faculty of the University Hospital Bonn and was conducted in accordance with the Declaration of Helsinki. All participants provided their written informed consent to participate in this study.

## Author Contributions

NM, DS, and RH designed the research. NM performed the research. NM, DS, and DP analyzed the data. NM, DS, DP, MO, and RH wrote the paper. All authors contributed to the article and approved the submitted version.

## Conflict of Interest

The authors declare that the research was conducted in the absence of any commercial or financial relationships that could be construed as a potential conflict of interest.
